# Supplementary material for: Circulating tumor cell detection in cancer patients using in-flow deep learning holography
Source: ArXiv. 2025 Jul 9:arXiv:2507.06536v1. Preprint. [Version 1] (PMC12265574)
Supplement: Supplement 1 [file NIHPP2507.06536v1-supplement-1.pdf]

Supplemental Information (SI)

1 Inertial Enrichment Chip

1.1 Intent & Purpose

The inertial enrichment step is necessary to reduce the overall number of cells seen by the system. The undiluted cell concentration in whole blood is extremely high – approximately 10^6 WBCs/mL and 10^9 RBCs/mL. The system was designed to work best when there is approximately 1 cell per hologram (thus ensuring an unambiguous fluorescence signal). An undiluted sample has thousands of cells in the 2.5 nL ROI, necessitating some form of dilution. This population is also dominated by RBCs which, while unlikely to be confused with CTCs, still contaminate the signal. Inertial enrichment was able to not only deplete RBCs, but to concentrate CTCs while retaining many of the WBCs. The enrichment ensured that CTCs formed a larger fraction of the sample, further reducing the impact of false positives.

1.2 Mechanism of Separation

As its name suggests, inertial enrichment separates cells primarily based on their mass, allowing for the removal of the much smaller RBCs. It also separates based on other mechanical properties such as deformability which enables the specialized concentration of CTCs.

Flow through curved channels can develop secondary motion in the form of counter-rotating vortices, known as Dean vortices. This has been observed to lead to the separation and even trapping of particles based on density. Repeated bends in a channel ultimately lead to spatial separation of the cells, enabling mechanical separation. For a full review on the topic, see Zhang et al. (2016).

1.3 Design Specifications

Inertial enrichment chips were fabricated from PDMS bonded to glass microscope slides. For further specifications on channel geometry, see patent (WO2024064911A1).

1.4 Cell Line Recovery

The retention of cancer cells processed through the inertial chip was validated with a mass-balanced recovery experiment. Approximately 3000 A549 cells were spiked into buffer media and run through the inertial device. The collection and waste outlets were collected and distributed to a well plate for counting. The experiment was repeated for 6 chips, each tested in triplicate. The recovery counts are shown in SI Table 1.

The median recovery is 95%. There were two replicates that failed with unusually low recovery. Generally, these failures are due to clogs or foreign material blocking the flow channel. When processing blood, these instances are readily identifiable by a visible change in the color of the collected sample due to an increase in the red blood cell concentration.

SI Table 1. Results of the inertial chip mass balance recovery experiments.

Chip ID	Replicate	Waste Count	Collect Count	Total Count	Recovery %
1	1	160	2704	2864	94%
1	2	143	3098	3241	96%
1	3	83	3029	3112	97%
2	1	90	3004	3094	97%
2	2	3320	14	3334	0%
2	3	94	3016	3110	97%
3	1	97	2877	2974	97%
3	2	155	3043	3198	95%
3	3	228	4621	4849	95%
4	1	83	2808	2891	97%
4	2	2975	52	3027	2%
4	3	51	3072	3123	98%
5	1	74	2806	2880	97%
5	2	299	2660	2959	90%
5	3	140	5535	5675	98%
6	1	187	2437	2624	93%
6	2	28	143	171	84%
6	3	213	3050	3263	93%

2 Data processing

2.1 PMT Signal

Raw fluorescence intensity signal captured by the photomultiplier tubes (PMTs) was preprocessed prior to peak detection. First, the timestamps associated with the PMT data were synchronized to the camera timestamps via periodic, controlled synchronization "events" that were readily detected in the PMT signal and the digital holographic microscopy (DHM) camera signal. Thus, any latency between the PMT and DHM data could be adjusted for when matching holographic model detections to spikes in fluorescence, to within a sub-frame capture timescale.

Fluorescent events were detected by first removing the background signal followed by convolution with a template signal, providing a "matched filter" for the target detection timescale. Peaks in the signal could then cleanly be detected via standard peak-finding algorithm based on prominence and width. PMT scoring provided an arbitrary but consistent scale for comparing detections against each other for fluorescence intensity. For patient samples, a PMT spike had to have a score > 8 to be identified as a PSMA positive cell.

2.2 Quality Control

Although the Detection Model was trained and augmented using a variety of image quality conditions, including variability in optical focus quality for cells, an automated method for assessing data quality was developed to ensure consistent results. Quantifying focus quality in holograms is notoriously difficult due to interference fringes that make standard metrics (e.g., sharpness) insufficiently robust. Instead, the cell focus was assessed indirectly using the holograms produced by small (sub-micron) debris particles that end up distributed randomly in the microfluidic channel. Debris on either side of the focal plane have a clearly distinct appearance and should be present in equal numbers around cells that are in focus. Using the same HRNet-based architecture as the Detection Model, a model for detecting such debris particles was developed from hand-labeled datasets to provide focus quality metric on a frame-by-frame basis. This focus quality metric was computed live on a separate server and made visible to the laboratory technician during data acquisition to ensure data quality consistency.

3 Deep Learning Model

3.1 Architecture

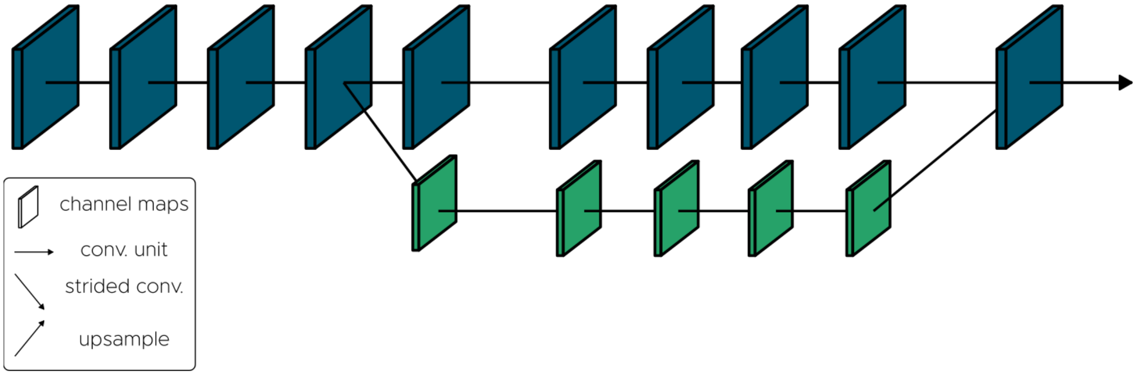

SI Fig. 1 Convolutional neural network used for real-time in-flow CTC detection.

The convolutional neural network (CNN) used by the DHM detection model was based on the original HRNet architecture proposed by Wang et al. (2020). To achieve the desired image processing throughput, the network was customized to the form shown in SI Fig. 1, after removing two levels of strided convolutions. The pixel-wise confidence heatmap output by this CNN was then used to detect cells of interest by applying a peak detection algorithm to yield confidence and position for each cell candidate.

3.2 Model validation

To supplement the System Validation results presented in the main text, we here present the false positive rate without the requirement that detections be IF positive. SI Fig. 2 corresponds to the same experimental data presented in Fig. 4b, where a false positive is any detection seen in the healthy samples.

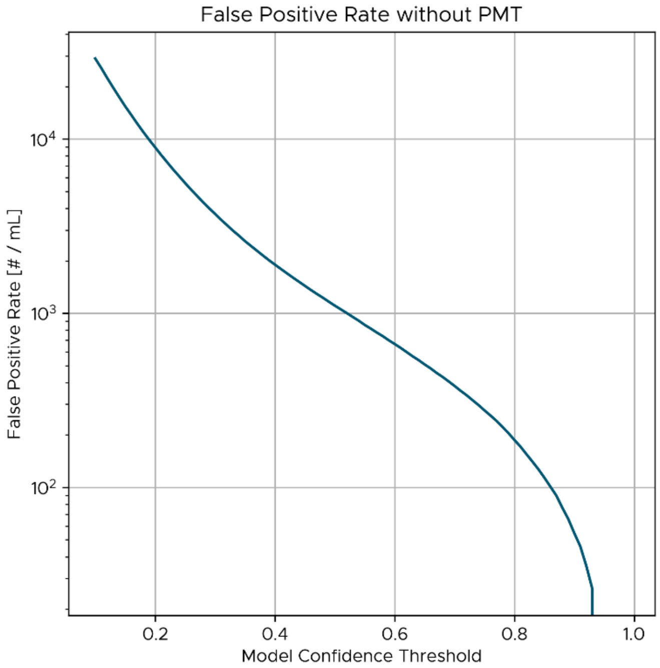

SI Fig. 2 DHM detection model (i.e., without integration of immunofluorescence signal captured with PMT) false positives for varying model confidence operating points from recovery experiment control samples.

4 Cell Lines and Blood Sample Examples

4.1 Image Examples

This section provides representative examples of both negative (healthy) and positive (cancer) cells, along with other objects encountered during model training. These examples illustrate the diverse morphological characteristics of CTCs and other cellular components found in blood samples.

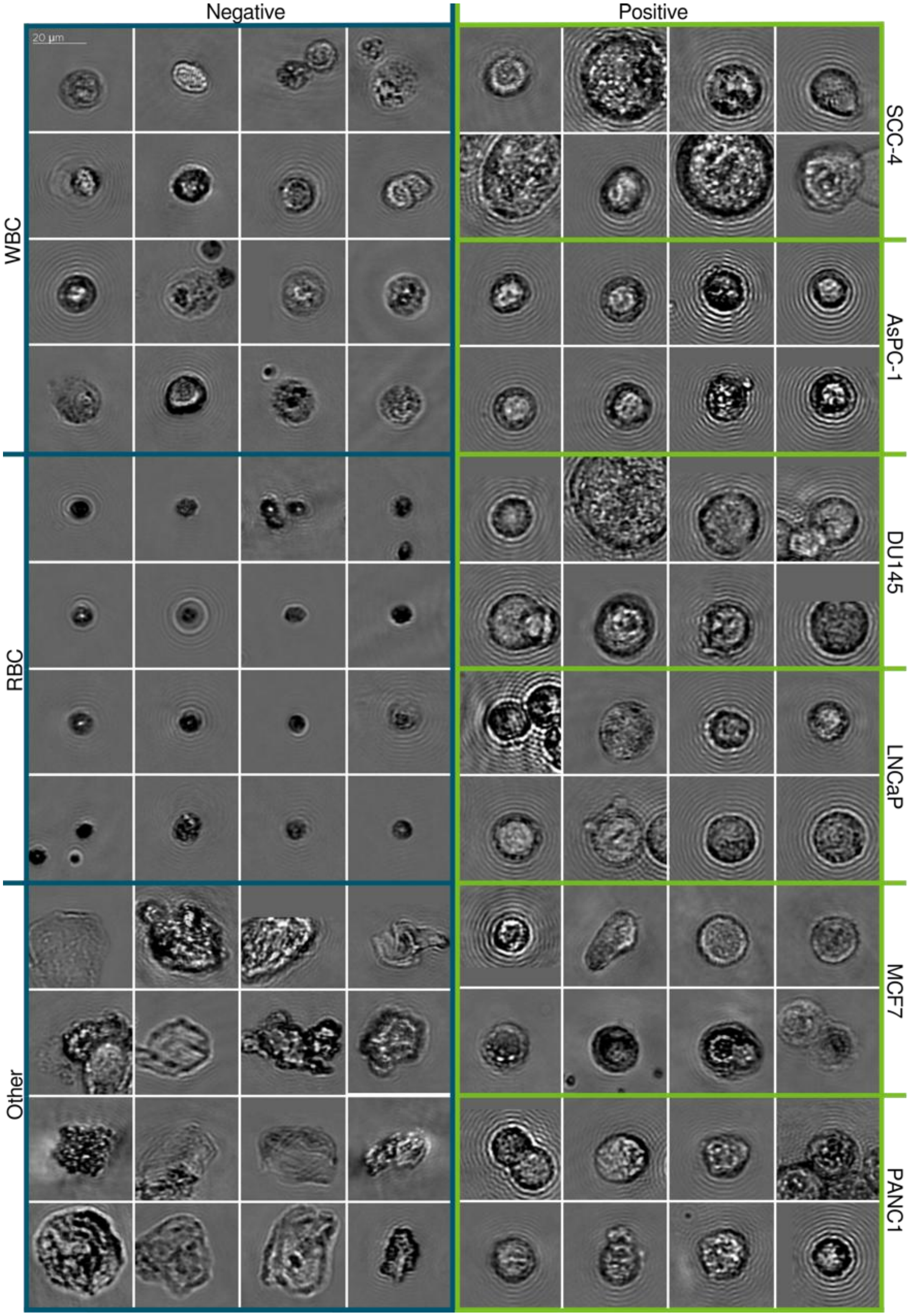

SI Fig. 3 Sample of negative (healthy) and positive (cancer) cells and objects seen during model development. Cell lines listed do not constitute an exhaustive list of all those used.

4.2 Cell Lines Used

SI Table 2. Cell lines used in model development and recovery data from Fig. 4a. Note that additional cell lines were used in theoretical recovery dataset, listed in SI Table 3.

<table>
<tr><th>Name</th><th>Cancer Tissue</th><th>Training</th><th>Model Validation</th><th>Experimental Recovery (Fig. 4a)</th><th>Theoretical Recovery (Fig. 4a)</th></tr>
<tr><td>AsPC-1</td><td>pancreas</td><td>X</td><td>X</td><td></td><td rowspan="6">X</td></tr>
<tr><td>DU 145</td><td>prostate</td><td>X</td><td>X</td><td></td></tr>
<tr><td>LNCaP</td><td>prostate</td><td>X</td><td>X</td><td>X</td></tr>
<tr><td>MCF7</td><td>breast</td><td>X</td><td>X</td><td></td></tr>
<tr><td>PANC1</td><td>pancreas</td><td>X</td><td>X</td><td></td></tr>
<tr><td>SCC4</td><td>oral</td><td></td><td>X</td><td></td></tr>
</table>

SI Table 3. Additional cell lines used in theoretical recovery (Fig. 4a)

Name	Cancer Tissue
A-375	melanoma
A-498	kidney
A-549	lung
AU-565	breast
BT-20	breast
BxPC-3	pancreas
CA-SK1	cervical
H-69	lung
HepG2	liver
Hs 578T	breast
MIA PaCa-2	pancreas
PC-3	prostate
SCC-01	oral
SCC-9	oral
SCC-15	oral
SCC-25	oral
SNU-16	gastric
T-24	bladder
T-84	colorectal
ZR-75-1	breast

Cell lines were chosen from a variety of cancers in order to maximize the diversity of cancer cell morphology that the model is trained on. True CTCs are rare, and therefore it is infeasible to train a supervised model on true CTC images. Thus, given the model's reliance on cell lines as a proxy, it was important for the model not to overfit to one particular cell line, as individual cell lines may not exactly match the morphological heterogeneity present in CTC populations. Therefore, multiple cancers are represented, with multiple cell lines for prostate cancer in particular, to hedge against this uncertainty.

5 Recovery Experiments

This section describes the methodology used to validate detection performance through controlled spiking experiments.

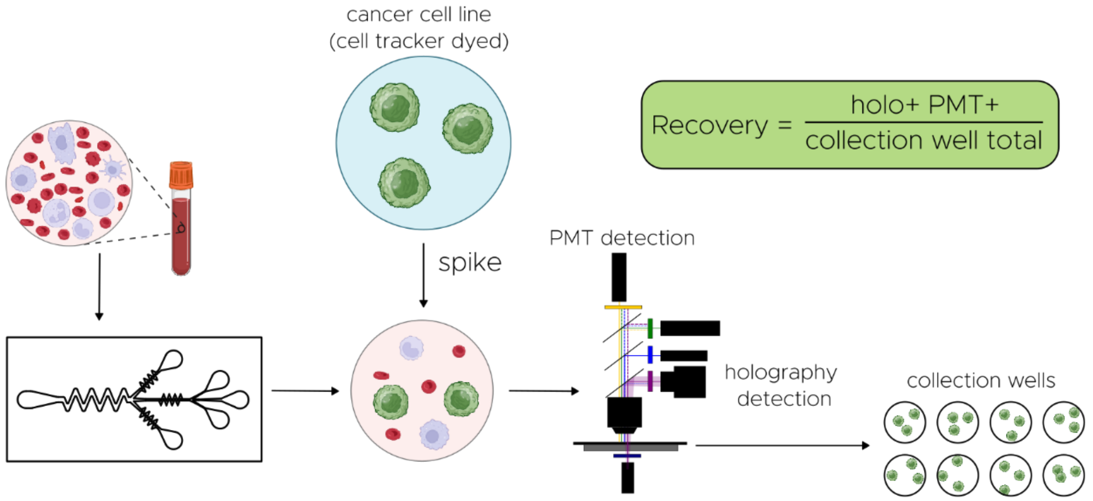

SI Fig 4. Spiking overview for recovery experiments for model performance validation.

5.1 Preparation of Spiked Samples

LNCaP cells were cultured in the same manner as those used for model training. These cells were stained with cell tracker green dye, prior to spiking into an enriched blood sample (i.e., after microfluidic depletion of RBCs and some WBCs). The cell tracker dye allowed for easy identification of spiked cells in the sample collected after imaging. Spiked cell numbers were targeted using a multi-step dilution procedure and confirmed by checking additional cell dilutions in a well plate counted under a fluorescent microscope.

5.2 Sample Processing & Analysis

Spiked blood samples were accompanied by control samples that did not have LNCaP cells spiked, from the same starting blood specimen. All samples, regardless of spiking, were pumped through a straight-channel microfluidic chip at the same flow rates as used for patient samples. The outlet volume of the sample was then transferred to a series of well plates and counted via a standard thresholding algorithm based on cell tracker dye expression. This method accounted for any discrepancies between the targeted spike number and the true spiked number, and enabled accurate measurement of the true

positives seen by the imaging system. Thus, the experimental recovery reflected the true model performance on this cell line.

Data collected via the PMTs were evaluated only on channel 1 (FITC equivalent), as there was only one stain used. Thus, PMT 2 was ignored as some of the cell tracker dye emissions bled into the second channel and PMT 2 was not expected to be negative.

5.3 Theoretical Recovery Analysis

The theoretical recovery was computed using a sample of 2 million cell line images collected during model training. Twenty additional cell lines were chosen but were not included in the training data set (see SI Table 3). Unlike the experimental spiked recovery, the theoretical recovery is estimated using the pseudo-labelled counts as the denominator. This difference in definition may account for the approximately 10% difference in the experimental and theoretical recovery curves (Fig. 4).

6 Patient and Healthy Sample Counts

This section provides a breakdown of the sample sizes, patient demographics, and key findings.

6.1 Cohort Characteristics

SI Table 4. Patient cohorts for blood samples used across model development and testing.

Cohort	No. of Individuals	Cancer Type	Gender	Source
Training Healthy	18	None	Male and female	Drawn from internal volunteers and purchased from ZenBio
Spike Validation	6	None	Male	Drawn from internal volunteers
Test Healthy	8	None	Male	Drawn from internal volunteers (n=2), Purchased from ZenBio (n=6)
Test Cancer	13	Prostate (Stage 4)	Male	Collected by collaborators at University of Minnesota

6.2 Per Patient CTC Enumeration Results

SI Table 5. Healthy patient and prostate cancer patient CTC counts.

Patient ID	Cancer Type	CTC count per mL
H01	Healthy	7.01
H02	Healthy	33.51
H03	Healthy	0.37
H04	Healthy	2.13
H05	Healthy	2.65
H06	Healthy	0.95
H07	Healthy	0.61
H08	Healthy	0.53
P01	Prostate	11.6
P02	Prostate	18.59
P03	Prostate	8.36
P04	Prostate	12.36
P05	Prostate	20
P06	Prostate	44.02
P07	Prostate	9.88
P08	Prostate	12.53
P09	Prostate	51.53
P10	Prostate	13.96
P12	Prostate	4.49
P12	Prostate	19.24
P13	Prostate	5.11

7 Station Description & Imaging Setup

7.1 System

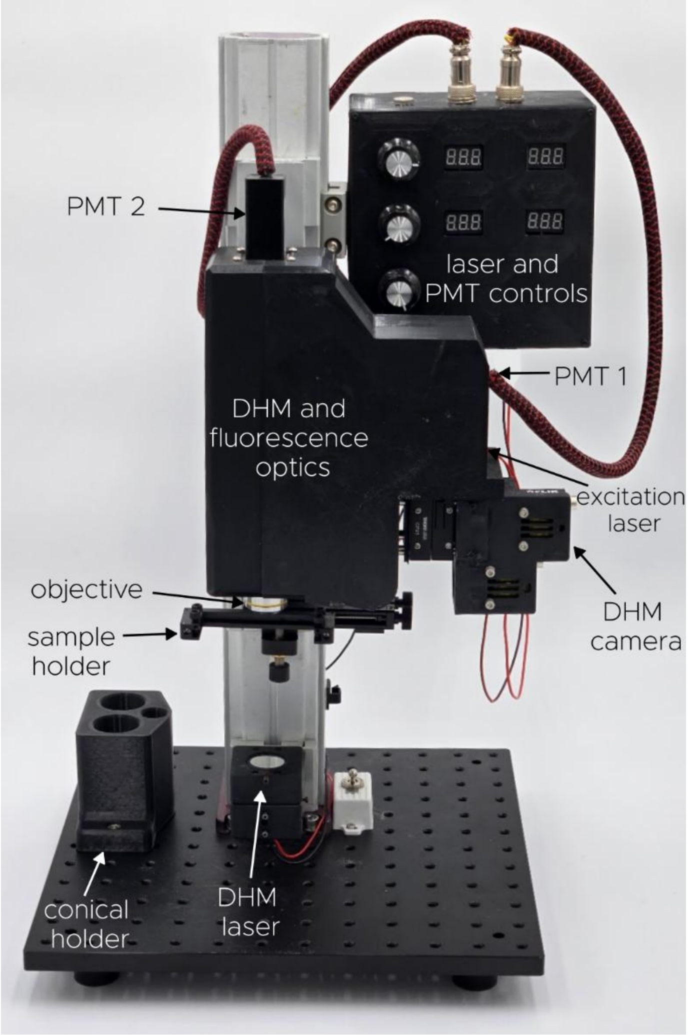

SI Fig. 5 Dual-modality imaging system, combining digital holographic microscopy (DHM) and fluorescence sensing with two photomultiplier tubes (PMTs).

7.2 Coherence & Stability Considerations

As a coherent imaging technique, DHM is highly sensitive to the quality of the illumination beam. The coherence length plays an important role – a more coherent source will improve the effective depth of focus of the system. This makes the station less sensitive to variations in the sample placement at a cost of increased noise from defects in optical components such as the dichroic mirrors and objective. Often, these defects can be removed by subtracting out a time-averaged background image. This enhancement further adds a requirement for high optical stability to ensure that the background is constant during the averaging period (approximately one second). The laser for this study was selected through

a process of trial and error where we qualitatively tested the image quality of several candidate lasers.

7.3 Fluorescence

Fluorescent excitation was induced using a single continuous laser diode with 488 nm center wavelength and 10 nm bandwidth. The laser beam was directed into the microscope objective (and then onto the sample) using a dichroic mirror with cut-on wavelength of 510 nm. This focused the laser onto the sample with a spot size measuring approximately 0.5 mm in diameter to induce fluorescent emission from labeled cells.

Photomultipliers tubes (PMTs) from Hamamatsu (H10722-20) were used to capture fluorescent emissions in a frequency bandwidth from DC to 20 kHz. Analog voltage signals produced by the low-noise amplifiers integrated into each PMT were captured on the same machine as the image acquisition via an analogue-to-digital converter (ADC). Each PMT captured emissions in one of two wavelengths: 525 nm, corresponding to the commonly used fluorescein isothiocyanate (FITC) channel, and 575 nm corresponding to the phycoerythrin (PE) channel. In each case, a combination of dichroic mirrors and bandpass optical filters were used to separate emissions reaching each PMT and to filter out light from the excitation laser and DHM laser. For the first PMT, capturing the FITC channel, a long-pass dichroic mirror with cut-on wavelength of 552 nm reflected shorter wavelength emissions through a 525 nm center wavelength bandpass filter with 25 nm full width-half max. For the second PMT, capturing the PE channel, longer wavelengths transmitted through the same 552 nm long-pass dichroic mirror were filtered through a 575 nm center wavelength bandpass filter with 25 nm full width-half max.

7.4 Microfluidics & Flow System

7.4.1 Chip Interface with Imaging System

Imaging was performed on a PDMS microfluidic chip similar to that used during microfluidic inertial enrichment (SI §1). For this application, sample was pumped along a straight channel and a sheath flow was also used to ensure all cells are concentrated in the center of the channel, away from channel walls which might otherwise produce image artifacts. Fluid was pumped through the channel using programmable syringe pumps (Pump Systems Inc., NE-500). The sample flow rate was 6 µL/min while the sheath flow rate was 7.5 µL/min.

During sample processing the microfluidic chip was mounted in a standard microscope slide holder (Thorlabs XYF1) allowing field-of-view (FOV) adjustments in the x-y plane, while focusing adjustment was enabled by a separate linear stage (Optics Focus MAX-B34C-13S) aligned with the z-axis. Alignment of the chip to the target FOV was performed manually using markers on the chip.

7.4.2 Photobleaching

The PDMS used for the microfluidic chip exhibited autofluorescence which may have adversely affected the SNR of true cell fluorescence peaks. To reduce this, we photobleached all chips for at least 1.5 hours prior to processing a sample. The bleaching

effectiveness was confirmed by measuring the decay of the autofluorescent signal (via the PMTs) over time. The chip was considered to be fully photobleached once the signal reached an asymptotic minimum.

7.5 System Footprint

The full system consisted of 3 connected components: the optical assembly (primary station), sample pumps, and computer. The optical assembly measured 30 cm x 30 cm x 60 cm (Width x Depth x Height) and sat on a lab bench. The sample syringe pumps were mounted on a shelf above the station while the acquisition computer (a standard desktop case) sat underneath the bench.

7.6 Automation & Control Software

A purpose-built software application (*HAIstack*) was built to operate the stations. This software allows the operator to document sample properties, view the live camera feed, control the pumps, and start and stop acquisition. This same interface also serves as the hub for the laboratory inventory management system (LIMS), including protocols, specimen database entries, data acquisition, session scheduling, etc.

The most labor-intensive aspects of the developing system were the assembly of the microfluidic chip (including inserting the sample, outlet, and waste tubes), alignment of the chip to the camera FOV, and monitoring for rare instances of leaks or other failures due to manufacturing defects. These processes can all be readily automated using robotics or through the design of specialized assembly hardware. Furthermore, the throughput of the system could be increased through the use of higher flow rates and corresponding imaging frame rates. The current system is bottlenecked by inference speeds at runtime, as only a subset of the acquired frames is saved, and used in later postprocessing, based on model confidence. Separating acquisition and inference is costlier in terms of the data pipeline required for storing images of the entire fluid volume, frame by frame, but would enable faster flow rates.

8 Supplementary References

Wang, J., Sun, K., Cheng, T., Jiang, B., Deng, C., Zhao, Y., ... & Xiao, B. (2020). Deep high-resolution representation learning for visual recognition. *IEEE Transactions on Pattern Analysis and Machine Intelligence*, 43(10), 3349-3364.

Zhang, J., Yan, S., Yuan, D., Alici, G., Nguyen, N. T., Warkiani, M. E., & Li, W. (2016). Fundamentals and applications of inertial microfluidics: A review. *Lab on a Chip*, 16(1), 10-34.
